# Application of Shear Wave Elastography in Assessing Peripheral Neuropathy in Parkinson’s Disease

**DOI:** 10.3390/brainsci16090988

**Published:** 2026-09-17

**Authors:** Yifan Song, Shuai Zheng, Lijuan Du, Hongxia Zhang, Wei Zhang, Wen He

**Affiliations:** 1 Department of Ultrasound, Beijing Tiantan Hospital, Capital Medical University, Beijing 100050, China; syf369788757@outlook.com (Y.S.); zheng.xiaoshuai@163.com (S.Z.); dulijuantt@163.com (L.D.); zhanghxttyy@sina.com (H.Z.); ultrazhangwei@126.com (W.Z.); 2 Department of Ultrasound, Beijing BOE Hospital Co., Ltd., Beijing 102402, China

**Keywords:** high-frequency ultrasound, Parkinson’s disease, peripheral neuropathy, shear wave elastography

## Abstract

**Highlights:**

**What are the main findings?**

- Shear wave velocity, measured in the longitudinal plane of the median nerve and in the common peroneal nerve, showed good intra- and inter-observer reproducibility and was significantly elevated in Parkinson’s disease patients with peripheral neuropathy.
- A combined model incorporating age, median nerve longitudinal shear wave velocity, and common peroneal nerve shear wave velocity achieved an area under the receiver operating characteristic curve of 0.938 for the diagnosis of Parkinson’s disease-related peripheral neuropathy.

**What are the implications of the main findings?**

- Longitudinal shear wave elastography provides a reliable, non-invasive imaging biomarker for the early detection of peripheral neuropathy in Parkinson’s disease, complementing conventional electrophysiological examinations.
- The multi-parameter diagnostic model can improve clinical decision-making by accurately identifying patients who may benefit from further neurological evaluation and early intervention.

**Abstract:**

Background/Objectives: Parkinson’s disease (PD) is a prevalent neurodegenerative disorder, and its comorbid peripheral neuropathy (PN) severely worsens patient prognosis. The diagnostic gold standard, nerve electrophysiological examination, has critical limitations, including invasiveness. This study aimed to explore the diagnostic value of high-frequency ultrasound combined with shear wave elastography (SWE) for PD-associated PN. Methods: Intra- and inter-observer reproducibility of ultrasound parameters (cross-sectional area [CSA], shear wave velocity [SWV]) of the median and common peroneal nerves was validated in 30 PD patients. A case–control study was conducted in 40 PD patients (stratified into PD-alone and PD-PN groups via the electrophysiological gold standard) and 40 healthy controls. Correlation analysis, logistic regression, and receiver operating characteristic (ROC) curve analysis were performed to assess diagnostic efficacy. Results: All measured parameters showed good reproducibility (intraclass correlation coefficient [ICC] > 0.855). Median nerve longitudinal SWV and common peroneal nerve SWV increased sequentially across the control, PD-alone, and PD-PN groups (after age adjustment using analysis of covariance (ANCOVA), *p* < 0.001), and showed weak-to-moderate statistically significant correlations with median nerve motor conduction latencies after Bonferroni correction for multiple comparisons. Age and common peroneal nerve SWV were identified as independent risk factors for PD-PN, while median nerve longitudinal SWV showed a positive but non-significant association in the multivariable model. A combined model incorporating age, median nerve longitudinal SWV, and common peroneal nerve SWV achieved an area under the ROC curve (AUC) of 0.938 (sensitivity 93.75%, specificity 91.67%; bootstrap-corrected AUC = 0.906). Conclusions: SWE-derived SWV has favorable diagnostic value for PD-associated PN with good reproducibility. The combination of age and multi-nerve SWV further improves diagnostic efficiency, providing a reliable non-invasive imaging biomarker for early PD-PN diagnosis. However, given the observational design and significant age imbalance between groups, residual confounding cannot be fully excluded, and prospective studies with age-matched cohorts are needed for definitive confirmation.

## 1. Introduction

Parkinson’s disease (PD) is the second most prevalent chronic neurodegenerative disorder worldwide, pathologically defined by progressive loss of dopaminergic neurons in the substantia nigra pars compacta and aberrant accumulation of α-synuclein-containing Lewy bodies [1]. Its core motor symptoms, including bradykinesia, resting tremor, muscular rigidity, and postural instability, lead to progressive impairment of activities of daily living, severely reduced quality of life, and a growing socioeconomic and healthcare burden globally [2]. Over the past three decades, the global prevalence of PD has more than doubled with the acceleration of population aging, and it has become a major public health challenge facing middle-aged and elderly populations worldwide [3].

Accumulating preclinical and clinical evidence has confirmed that the peripheral nervous system is a key early target of α-synuclein pathological damage in PD, and peripheral neuropathy (PN) has been increasingly recognized as one of the most common non-motor complications of the disease. Depending on the diagnostic criteria and electrophysiological assessment methods adopted, the reported prevalence of PN in PD patients ranges widely from 30% to 55% [4,5]. PD-associated PN not only exacerbates gait abnormalities, sensory deficits, and balance dysfunction, but also significantly increases the risk of falls and fractures in patients, and has been identified as an independent predictor of poor long-term prognosis and reduced survival in PD cohorts [6,7]. Notably, recent studies have revealed that small-fiber neuropathy (SFN) may already occur in the early stage of PD, even before the initiation of dopaminergic replacement therapy, which indicates that peripheral nerve involvement is an inherent pathological manifestation of PD, rather than a treatment-related adverse event alone [8]. This further highlights the urgent clinical need for early identification and dynamic monitoring of peripheral nerve damage in PD patients.

At present, nerve conduction studies (NCS) combined with electromyography are the clinical gold standard for the diagnosis and classification of PD-associated PN. Although NCS can objectively evaluate the function of large myelinated nerve fibers, it has notable inherent limitations in clinical practice [9]. First, NCS is an invasive and time-consuming examination, with poor compliance in elderly PD patients complicated with cognitive impairment, motor dysfunction or severe tremor. Second, its results are highly operator-dependent and susceptible to interference from patient age, body mass index, and limb temperature, with non-negligible false-positive and false-negative rates in clinical application. Most importantly, NCS has extremely limited sensitivity for detecting early subclinical nerve injury and SFN, and it cannot provide direct visualized information on the structural pathological changes of peripheral nerves, such as axonal loss, demyelination, or endoneurial fibrosis [10]. These limitations greatly restrict its application in early screening, routine follow-up and dynamic monitoring of PD-associated PN, and underscore the urgent need to develop a non-invasive, repeatable, and quantitative imaging modality to complement and optimize the existing electrophysiological assessment system.

High-frequency ultrasound (HFUS) has emerged as a reliable non-invasive imaging tool for peripheral nerve evaluation in clinical practice. It can achieve real-time, high-resolution visualization of the morphological structure, course, echotexture, and adjacent tissue relationship of peripheral nerves, and can quantitatively measure the cross-sectional area (CSA) of nerves [11]. However, CSA measurement alone can only reflect the morphological enlargement of nerves after obvious pathological changes, and often fails to detect early subclinical structural alterations before overt nerve swelling occurs, which limits its value in early diagnosis of PN. As an advanced quantitative extension of conventional ultrasound technology, shear wave elastography (SWE) can non-invasively assess the stiffness of biological tissues by measuring shear wave velocity (SWV), which is directly proportional to the elastic modulus of the tissue and can objectively reflect histopathological changes caused by nerve injury [12]. Previous studies have confirmed that increased nerve stiffness, as reflected by elevated SWV, is a common pathological manifestation of peripheral nerve damage of various etiologies. The diagnostic value of SWE has been widely verified in diabetic peripheral neuropathy (the most common secondary PN) and carpal tunnel syndrome, and SWV has been found to be significantly correlated with the degree of axonal loss, demyelination and endoneurial fibrosis [13,14].

Nevertheless, the application of SWE in the evaluation of PD-associated PN remains largely unexplored to date. Most existing SWE studies have focused on diabetic peripheral neuropathy, while the few available reports on PD-associated PN are limited by small sample sizes, lack of rigorous reproducibility validation of standardized measurement protocols, and inconsistent nerve sites and scanning planes selected [15]. Furthermore, peripheral nerves have significant anisotropic biomechanical properties due to the parallel arrangement of nerve fiber bundles, which means that the stiffness measured in the longitudinal plane (parallel to nerve fibers) and cross-sectional plane may reflect different levels of pathological information. It remains unclear whether longitudinal SWV has superior diagnostic performance compared with cross-sectional SWV in the detection of PD-associated PN, and whether combining SWV parameters from multiple commonly affected nerves with clinical characteristics can further improve the diagnostic accuracy for PD-associated PN. These unresolved key scientific issues have become an important barrier to the clinical translation of SWE in the PD population.

Therefore, the present study systematically carried out three-stage experiments to address the above research gaps, with the following specific aims: (1) to evaluate the intra- and inter-observer reproducibility of HFUS combined with SWE for measuring CSA and SWV of the median nerve (forearm segment) and common peroneal nerve (fibular head segment) in PD patients, so as to establish a standardized measurement protocol; (2) to compare the differences in nerve CSA and SWV values among healthy controls, PD patients without PN, and PD patients with PN (diagnosed by NCS gold standard); (3) to analyze the correlation between SWV parameters and gold-standard electrophysiological indicators, so as to clarify the pathophysiological significance of SWE measurements; and (4) to construct and validate a non-invasive predictive model incorporating age and multi-nerve SWV parameters, to optimize the non-invasive diagnosis of PD-associated PN. We hypothesized that the longitudinal SWV of the median nerve and the SWV of the common peroneal nerve would be significantly elevated in PD patients with PN, and could serve as independent imaging biomarkers for the early detection of PD-associated PN.

## 2. Materials and Methods

### 2.1. Study Design and Ethical Approval

This study was a single-center, prospective case–control study, conducted in strict accordance with the Declaration of Helsinki and the Strengthening the Reporting of Observational Studies in Epidemiology (STROBE) guidelines. The study protocol was approved by the Institutional Review Board of Beijing Tiantan Hospital Affiliated to Capital Medical University (protocol code: KY2022-015-04; approval date: 15 April 2022). All subjects were fully informed of the study content before enrollment, and signed written informed consent for participation and for the publication of anonymized study data.

### 2.2. Study Subjects

Two cohorts of subjects were consecutively enrolled in this study from May 2022 to August 2023 at Beijing Tiantan Hospital Affiliated to Capital Medical University:

1. Reproducibility validation cohort: 30 patients with confirmed PD were enrolled to evaluate the intra- and inter-observer reliability of ultrasound measurements.
2. Case–control study cohort: 40 consecutive patients with confirmed PD were enrolled as the case group, and 40 age- and sex-matched healthy volunteers were enrolled as the healthy control group during the same period.

Inclusion criteria for PD patients were as follows: (1) aged 30–85 years; (2) definite diagnosis of PD in accordance with the UK Parkinson’s Disease Society Brain Bank clinical diagnostic criteria; (3) ability to cooperate with all relevant examinations; and (4) voluntary participation in the study with signed informed consent.

Inclusion criteria for healthy controls were as follows: (1) age- and sex-matched with the PD group; (2) no neurological symptoms or signs related to PN; (3) normal results in NCS; (4) no contraindications for ultrasound examination; and (5) voluntary participation in the study with signed informed consent.

Exclusion criteria (applicable to both cohorts): (1) complicated with PN caused by other etiologies, including diabetes mellitus, impaired glucose tolerance, cervical/lumbar radiculopathy, cerebral infarction, Guillain-Barré syndrome, toxic neuropathy, hereditary neuropathy, vitamin B deficiency, and thyroid dysfunction; (2) complicated with severe peripheral arteriovenous vascular disease that may affect nerve blood supply and ultrasound imaging quality; (3) history of peripheral nerve trauma or surgery, or nerve damage caused by drug-induced neurotoxicity or metabolite accumulation due to renal insufficiency; (4) poor two-dimensional ultrasound image quality due to excessive subcutaneous fat or limb deformity, which made reliable SWE measurement impossible; and (5) unable to cooperate with examinations due to severe cognitive dysfunction, language communication disorder, or acute mental illness.

Using NCS as the gold standard [9], the 40 PD patients in the case–control cohort were divided into two subgroups: the PD without PN group (PD-alone group, *n* = 24) and the PD with PN group (PD-PN group, *n* = 16).

### 2.3. Ultrasound Examination and Reproducibility Validation

#### 2.3.1. Instrumentation

All ultrasound examinations were performed using the same Canon Aplio i900 color Doppler ultrasound diagnostic instrument (Canon Medical Systems, Otawara, Japan) equipped with the system’s built-in quantitative SWE software (a factory-installed, device-integrated application without a standalone release version; the software/hardware platform is identified by the Canon Aplio i900 instrument model) and an i18LX5 high-frequency linear array probe (frequency range: 5–18 MHz). The instrument parameters were fixed throughout the study period to avoid systematic errors.

#### 2.3.2. Standardized Examination Protocol

Before examination, all subjects rested in a temperature-controlled examination room (22–24 °C) for at least 15 min to ensure the skin temperature of the examined limb was stabilized at 32 ± 1 °C, eliminating the interference of temperature on nerve stiffness and imaging quality. During the examination, the probe was kept in light contact with the skin without applying any additional pressure to the target nerve, to avoid artificial changes in tissue stiffness.

Median nerve examination: The subject was placed in the supine position, with the upper limb abducted less than 90°, the forearm relaxed, the palm facing up, and the wrist joint in a neutral position. Cross-sectional scanning of the median nerve was performed at 4–5 cm proximal to the wrist crease. The image was frozen when the characteristic “sieve-like” hyperechoic structure of the median nerve was clearly displayed on two-dimensional ultrasound. The CSA of the median nerve was measured by manually tracing along the inner edge of the nerve epineurium, with 3 repeated measurements for each side and the average value taken. The SWE mode was then activated, and after the elastogram was stabilized with uniform color filling, a circular region of interest (ROI) with a diameter of 3 mm was placed in the center of the nerve parenchyma, avoiding the epineurium and surrounding tissues. The SWV was measured 3 times repeatedly in the cross-section, and the average value was recorded. The probe was rotated 90° in situ to obtain a longitudinal section parallel to the course of the median nerve fibers. After the parallel linear fiber bundle structure of the nerve was clearly displayed, the longitudinal SWV was measured 3 times with the same ROI settings, and the average value was recorded.

Common peroneal nerve examination: The subject was placed in the lateral decubitus position (examined limb on the upper side, knee joint flexed at 30°) or prone position. Cross-sectional scanning of the common peroneal nerve was performed posterior to the fibular head. The image was frozen when the flat oval “sieve-like” hyperechoic structure of the common peroneal nerve was clearly displayed. The CSA and cross-sectional SWV of the common peroneal nerve were measured using the same protocol, ROI settings, and quality control standards as the median nerve examination.

All subjects completed bilateral examinations of the median nerve and common peroneal nerve following the above standardized protocol. All ultrasound examinations were performed by the same sonographer who received standardized training in peripheral nerve ultrasound and SWE. All ultrasound examinations in the case–control cohort were performed by a sonographer who was blinded to the participants’ clinical diagnosis and NCS results; NCS was conducted by a separate neurophysiologist, and the results were not disclosed to the sonographer until after all ultrasound data were acquired and locked. Representative cases for figure display were selected based on proximity to the group mean (within ±0.5 m/s of the respective group mean SWV) to ensure that the illustrative images faithfully represent the typical findings for each group.

#### 2.3.3. Reproducibility Validation

For the 30 PD patients in the reproducibility validation cohort, measurements were independently performed by two sonographers with different seniority in a blinded manner to evaluate inter-observer reliability: Sonographer A (with more than 5 years of experience in peripheral nerve ultrasound and SWE) completed the first measurement; Sonographer B (with 1 year of relevant experience) completed the second measurement at the same anatomical site within 2 h, without access to the first measurement results or the subject’s clinical data. To evaluate intra-observer reliability, Sonographer A performed a third measurement 24 h after the first measurement, without referring to the previous results. Both sonographers were blinded to the subject’s NCS results and clinical information throughout the validation process.

#### 2.3.4. Image Quality Control Criteria

The quality control criteria for SWE measurement were as follows: the effective filling area of the elastic sampling frame was ≥50% of the total ROI area, the minimum SWV in the ROI was >0 m/s, and there was no obvious artifact. Images that did not meet the criteria were re-scanned and measured immediately until qualified images were obtained. The intraclass correlation coefficient (ICC) was used to evaluate measurement reliability: ICC < 0.5 indicated poor reliability, 0.5 ≤ ICC < 0.75 indicated moderate reliability, 0.75 ≤ ICC < 0.9 indicated good reliability, and ICC ≥ 0.9 indicated excellent reliability.

### 2.4. Gold Standard: Nerve Conduction Studies

All subjects in the case–control cohort completed bilateral upper and lower limb NCS within 1 week of the ultrasound examination, using a Keypoint 9 electromyography and evoked potential instrument (Dantec, Skovlunde, Denmark). The examination was performed in a shielded room with a room temperature of 22–24 °C, and the limb skin temperature was maintained above 32 °C throughout the examination. All NCS procedures were completed by the same neurophysiologist with more than 10 years of experience, who was blinded to the subject’s ultrasound examination results and clinical grouping.

The examination included the following parameters:

1. Median nerve: Motor conduction study (stimulation at the wrist and elbow, recording the compound muscle action potential (CMAP) from the abductor pollicis brevis muscle, with measurement of latency, amplitude, and conduction velocity); orthodromic sensory conduction study (stimulation at the third finger, recording the sensory nerve action potential (SNAP) at the wrist, with measurement of latency, amplitude, and conduction velocity).
2. Common peroneal nerve: Motor conduction study (stimulation at the ankle, below the fibular head, and above the fibular head, recording the CMAP from the extensor digitorum brevis muscle, with measurement of latency, amplitude, and conduction velocity); orthodromic sensory conduction study (stimulation at the dorsolateral foot, recording the SNAP above the lateral malleolus, with measurement of latency, amplitude, and conduction velocity).

The diagnostic criteria for PD-associated PN (gold standard) were formulated in accordance with the American Academy of Neurology (AAN) guidelines for the diagnosis of PN [9]: the presence of at least 2 abnormal electrophysiological parameters in at least 1 nerve, including decreased motor/sensory conduction velocity, prolonged latency, or decreased amplitude, consistent with electrophysiological manifestations of axonal injury or demyelination.

### 2.5. Statistical Analysis

All statistical analyses were performed using SPSS 25.0 (IBM Corp., Armonk, NY, USA) and MedCalc 20.0 (MedCalc Software Ltd., Ostend, Belgium). A two-sided *p* value < 0.05 was considered statistically significant.

1. Normality test and data description: The Shapiro–Wilk test was used to test the normality of all measurement data. Normally distributed measurement data were presented as mean ± standard deviation ($\bar{x}$ ± *s*), and non-normally distributed data were presented as median (interquartile range) [M(P25, P75)]. Count data were presented as frequency (percentage). The Shapiro–Wilk test confirmed normality for all SWV and CSA parameters across all three groups (all *p* > 0.05); a Kruskal–Wallis non-parametric sensitivity analysis yielded consistent results.
2. Reliability analysis: A two-way mixed-effects model with absolute agreement definition was used to calculate ICC and 95% confidence interval (CI) for intra- and inter-observer reliability evaluation.
3. Intergroup comparison: Paired *t*-test was used to compare the differences in ultrasound parameters between the bilateral nerves of the same subject. The ICC between left and right measurements was calculated, and per-subject averages of bilateral measurements were used as the primary unit of analysis for all group comparisons. Analysis of covariance (ANCOVA) with age as a continuous covariate was used [16] for comparison of SWV parameters among the three groups, with Bonferroni post hoc test for pairwise comparison. One-way ANOVA was used for CSA comparisons. Chi-square test (χ^2^ test) or Fisher’s exact test was used for comparison of count data among groups.
4. Correlation analysis: Spearman rank correlation analysis was used to explore the correlation between nerve SWV parameters and NCS electrophysiological parameters, with correlation coefficient r and *p* value calculated. To account for the within-subject correlation between bilateral measurements, a linear mixed-effects model with a random intercept for each subject was used to adjust the *p* values. Bonferroni correction was applied to account for multiple comparisons (α′ = 0.05/8 = 0.00625) [17].
5. Risk factor analysis and diagnostic model construction: All diagnostic model analyses (logistic regression, ROC curve, bootstrap internal validation, calibration assessment, and decision curve analysis) were performed at the subject level using PD patients only (16 PD-PN vs. 24 PD-alone), excluding healthy controls, because the model is intended to diagnose PN among patients with already confirmed PD. Variables with *p* < 0.05 in univariate analysis were included in multivariate binary logistic regression analysis with the enter (forced-entry) method, in which all three pre-specified predictors (age, median nerve longitudinal SWV, common peroneal nerve SWV) were entered simultaneously, to identify independent risk factors for PD-associated PN, and odds ratio (OR) and 95% CI were calculated. A combined diagnostic prediction model was constructed based on the independent risk factors. Receiver operating characteristic (ROC) curves were plotted to calculate the area under the curve (AUC), sensitivity, specificity, and Youden index, to evaluate the diagnostic efficacy of single parameters and the combined model. Bootstrap internal validation with 1000 resampling iterations was performed to assess the optimism-corrected AUC of the combined model [18]. Calibration was assessed using the Brier score and calibration slope, and clinical utility was evaluated by decision curve analysis (DCA) [19].
6. Sample size calculation: Based on previously reported diagnostic efficacy of SWE for PN, with a two-sided α = 0.05, power (1 − β) = 0.8, expected AUC of 0.80, and a 1:1 ratio between the control group and the case group, the sample size calculation was performed using PASS 15.0 Power Analysis and Sample Size software (NCSS, LLC., Kaysville, UT, USA). The result showed that at least 32 subjects were required in each group. The 40 PD patients and 40 healthy controls enrolled in this study met the sample size requirement for the primary PD-versus-control comparison. The subsequent three-group stratification (PD-alone *n* = 24, PD-PN *n* = 16) and diagnostic model construction (events-per-variable (EPV) = 16/3 ≈ 5.3, below the conventional minimum of 10 [20]) were secondary exploratory analyses with reduced statistical power, and the risk of overfitting is acknowledged in the Limitations.

## 3. Results

### 3.1. General Characteristics and Baseline Comparisons Among Groups

A total of 80 subjects were enrolled in this study, including 40 patients with PD and 40 healthy volunteers as the control group. Using nerve electrophysiological examination as the reference standard, PD patients were further divided into the PD-alone group (*n* = 24) and the PD-PN group (*n* = 16).

No significant differences were observed in gender distribution (χ^2^ = 0.802, *p* = 0.670), height (F = 2.044, *p* = 0.133), or body weight (F = 1.512, *p* = 0.232) among the three groups. A statistically significant difference in age was detected across groups (F = 7.893, *p* < 0.001). Post hoc pairwise comparisons revealed that the mean age of the PD-PN group [(67.56 ± 11.03) years] was significantly higher than that of the PD-alone group [(56.88 ± 10.39) years] and the control group [(56.78 ± 15.60) years] (both *p* < 0.001), while no significant age difference existed between the PD-alone and control groups (*p* = 0.976).

### 3.2. Reproducibility and Reliability of Ultrasound Measurements

Inter-observer reliability analysis demonstrated that the ICC values for median nerve CSA, median nerve longitudinal SWV, common peroneal nerve CSA, and common peroneal nerve SWV were all >0.855, indicating good to excellent reliability according to the Landis–Koch criteria for measurements performed by ultrasound physicians with different seniority levels.

Intra-observer reliability analysis also showed that all ICC values for two independent measurements by Examiner A were >0.865, confirming excellent reproducibility of the measurement protocol (Table 1).

### 3.3. Comparisons of Ultrasound Parameters Among Groups

No statistically significant differences were found in the CSA or SWV of the median and common peroneal nerves between the left and right sides in any of the three groups (all *p* > 0.05, paired *t*-test). The ICC between left and right measurements was 0.78 for median nerve longitudinal SWV and 0.74 for common peroneal nerve SWV, indicating substantial within-subject correlation. Therefore, per-subject averages of bilateral measurements were used for all subsequent group comparisons and regression analyses.

The Shapiro–Wilk test confirmed normality for all SWV and CSA parameters across all three groups (*p* values ranged from 0.156 to 0.412; all *p* > 0.05), including the smallest subgroup (PD-PN, *n* = 16) where all *p* values exceeded 0.19. A Kruskal–Wallis non-parametric sensitivity analysis yielded consistent results (median nerve longitudinal SWV: *p* < 0.001; common peroneal nerve SWV: *p* < 0.001), confirming the robustness of the parametric findings.

**Figure 1:**
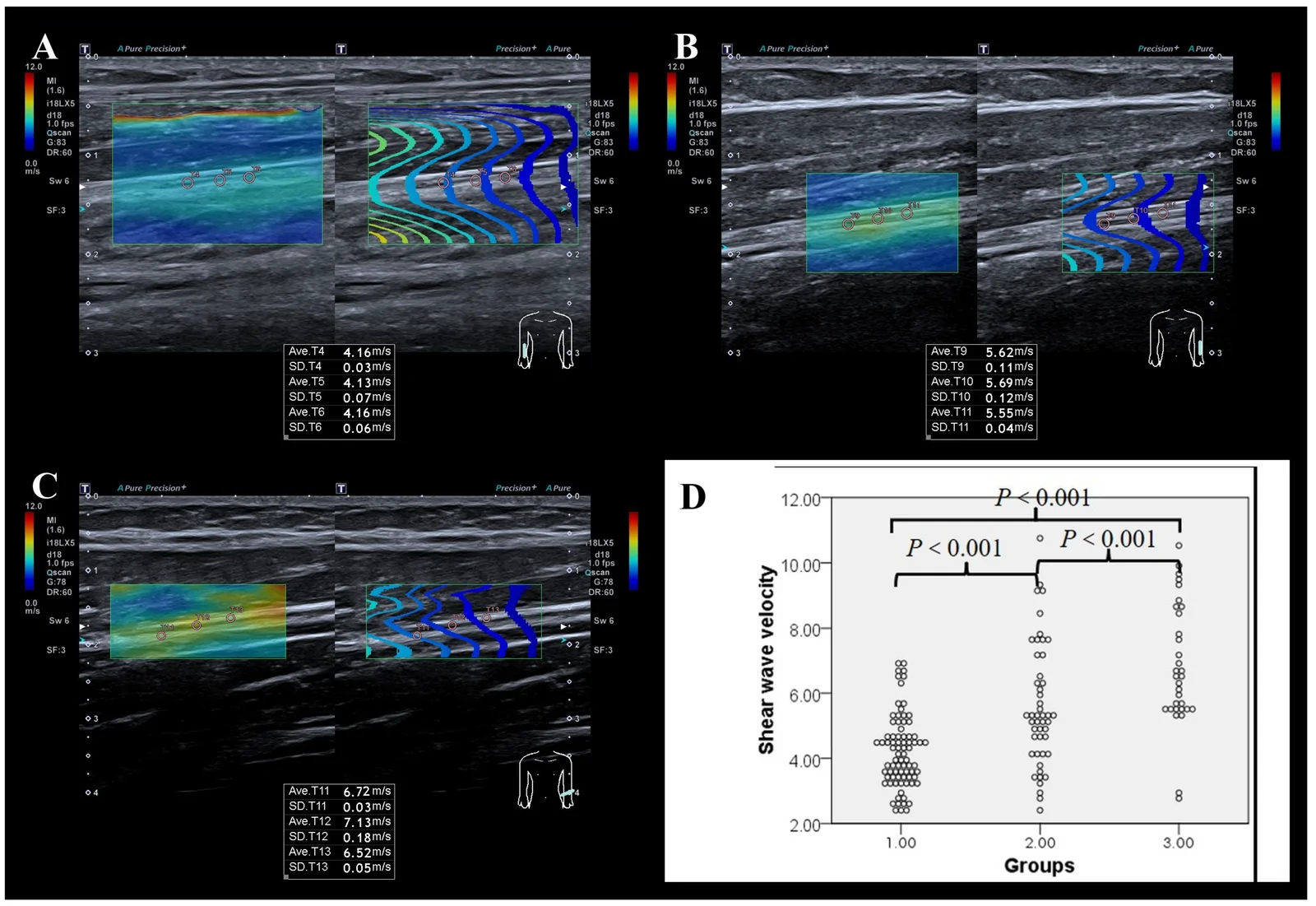
Longitudinal SWV of the median nerve in the three groups. Panels (**A**–**C**) show representative individual cases (selected within ±0.5 m/s of the group mean): (**A**) Control group, SWV = 4.15 m/s (group mean: 4.219 m/s). (**B**) PD-alone group, SWV = 5.62 m/s (group mean: 5.584 m/s). (**C**) PD-PN group, SWV = 6.79 m/s (group mean: 6.838 m/s). (**D**) Age-adjusted ANCOVA results of SWV among the three groups.

No significant intergroup differences were observed in median nerve CSA (*p* > 0.05). Median nerve cross-sectional SWV showed a non-significant increasing trend across groups, with values of (4.187 ± 1.105) m/s, (5.476 ± 1.854) m/s, and (6.712 ± 1.723) m/s for the control, PD-alone, and PD-PN groups, respectively (F = 2.876, *p* = 0.062). Median nerve longitudinal SWV increased progressively from the control group to the PD-alone group and then to the PD-PN group, with values of (4.219 ± 1.117) m/s, (5.584 ± 1.893) m/s, and (6.838 ± 1.897) m/s, respectively. After adjustment for age using ANCOVA, the overall difference among groups remained statistically significant (F = 22.874, *p* < 0.001, Figure 1). The age-adjusted estimated marginal means (least-squares means) were 4.312 m/s (control), 5.498 m/s (PD-alone), and 6.547 m/s (PD-PN), preserving the progressive increasing trend across groups. Post hoc Bonferroni tests confirmed significant differences between all pairwise comparisons (all adjusted *p* < 0.05).

**Figure 2:**
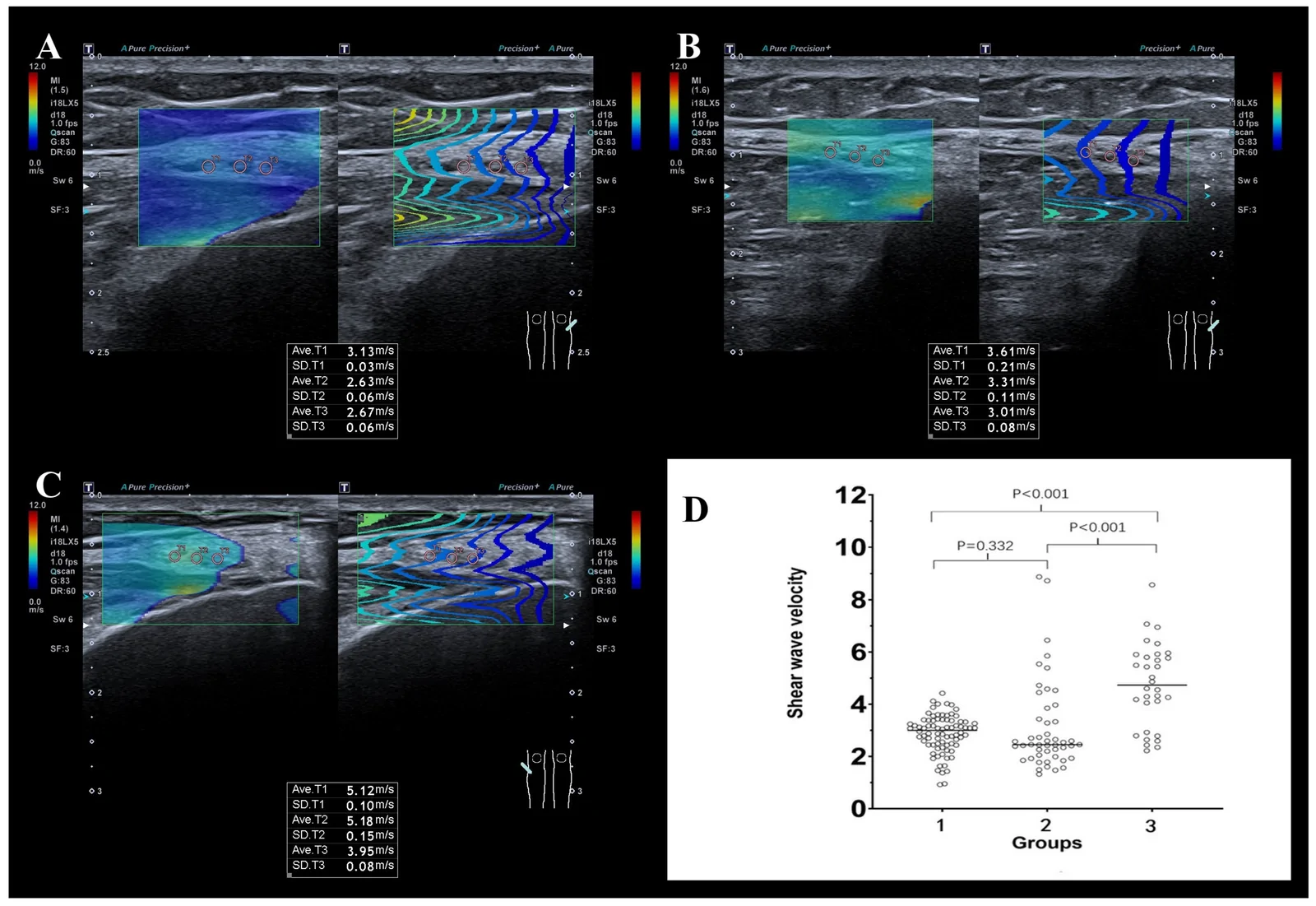
SWV of the common peroneal nerve in the three groups. Panels (**A**–**C**) show representative individual cases (selected within ± 0.5 m/s of the group mean): (**A**) Control group, SWV = 2.87 m/s (group mean: 2.893 m/s). (**B**) PD-alone group, SWV = 3.31 m/s (group mean: 3.277 m/s). (**C**) PD-PN group, SWV = 4.75 m/s (group mean: 4.778 m/s). (**D**) Age-adjusted ANCOVA results of SWV among the three groups.

Similarly, no significant difference in common peroneal nerve CSA was detected among the three groups (*p* > 0.05). Common peroneal nerve SWV also showed a progressively increasing trend across groups, with values of (2.893 ± 0.760) m/s, (3.277 ± 1.620) m/s, and (4.778 ± 1.662) m/s for the control, PD-alone, and PD-PN groups, respectively. After adjustment for age using ANCOVA, the overall intergroup difference remained statistically significant (F = 11.536, *p* < 0.001, Figure 2). The age-adjusted estimated marginal means were 2.956 m/s (control), 3.312 m/s (PD-alone), and 4.521 m/s (PD-PN). Post hoc Bonferroni tests showed that the PD-PN group had significantly higher SWV than both the PD-alone and control groups (both adjusted *p* < 0.01), while no significant difference was found between the PD-alone and control groups.

### 3.4. Correlations Between Ultrasound Parameters and Nerve Electrophysiological Parameters

**Figure 3:**
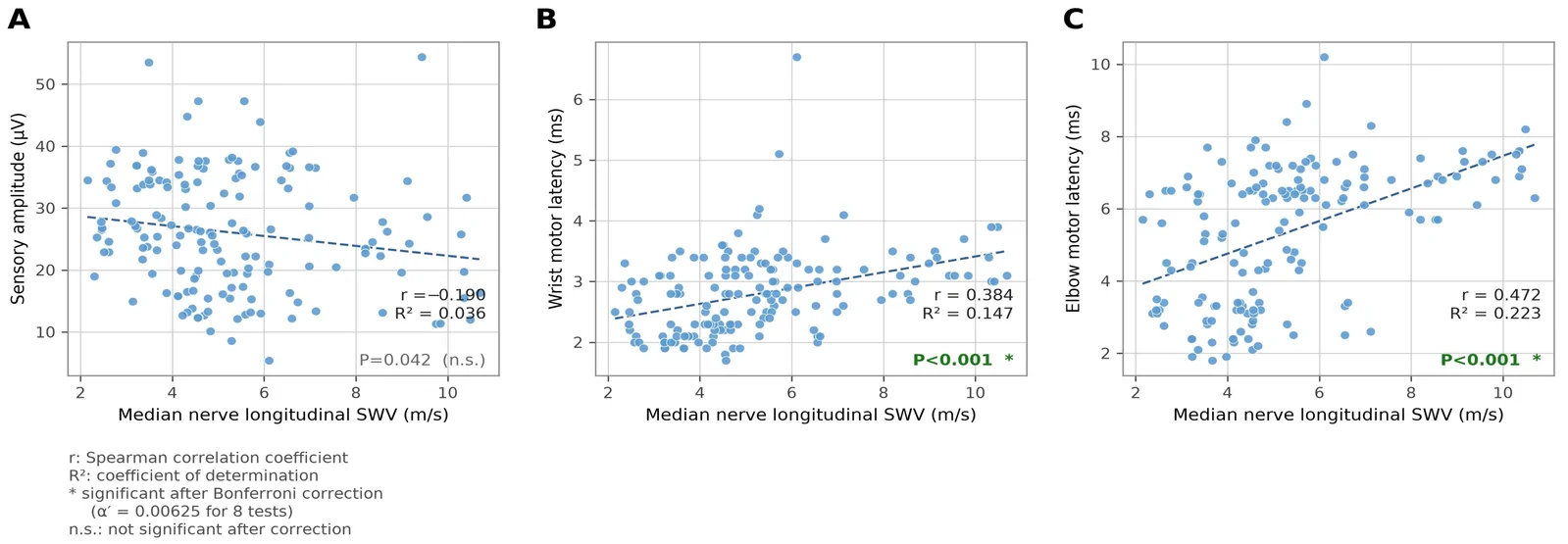
Correlation analysis between median nerve longitudinal SWV and nerve electrophysiological parameters. (**A**) Negative correlation between SWV and sensory nerve conduction (SCV) peak-to-peak amplitude (μV) of the median nerve (finger III–wrist). (**B**) Positive correlation between SWV and motor nerve conduction (MCV) wrist latency (ms) of the median nerve. (**C**) Positive correlation between SWV and MCV elbow latency (ms) of the median nerve. Light blue dots represent individual observations, and the dark blue dashed line denotes the linear regression fit; *p* values shown in green indicate significance after Bonferroni correction, whereas gray *p* values denote non-significant (n.s.) correlations. The symbols r, R^2^, *, and n.s. are defined in the figure legend.

Spearman rank correlation analysis (with *p* values adjusted via a linear mixed-effects model accounting for within-subject correlation) showed that median nerve longitudinal SWV was negatively correlated with the sensory nerve conduction peak-to-peak amplitude of the median nerve (finger III–wrist) (*r* = −0.190, *p* = 0.042), and positively correlated with the wrist latency (*r* = 0.384, *p* < 0.001) and elbow latency (*r* = 0.472, *p* < 0.001) of median nerve motor conduction (Figure 3). After Bonferroni correction for multiple comparisons (α′ = 0.00625), only the correlations with motor conduction latencies (wrist and elbow) remained statistically significant (*r* = 0.384, R^2^ = 0.147 for wrist latency; *r* = 0.472, R^2^ = 0.223 for elbow latency); the correlation with sensory amplitude did not survive correction (*r* = −0.190, R^2^ = 0.036).

**Figure 4:**
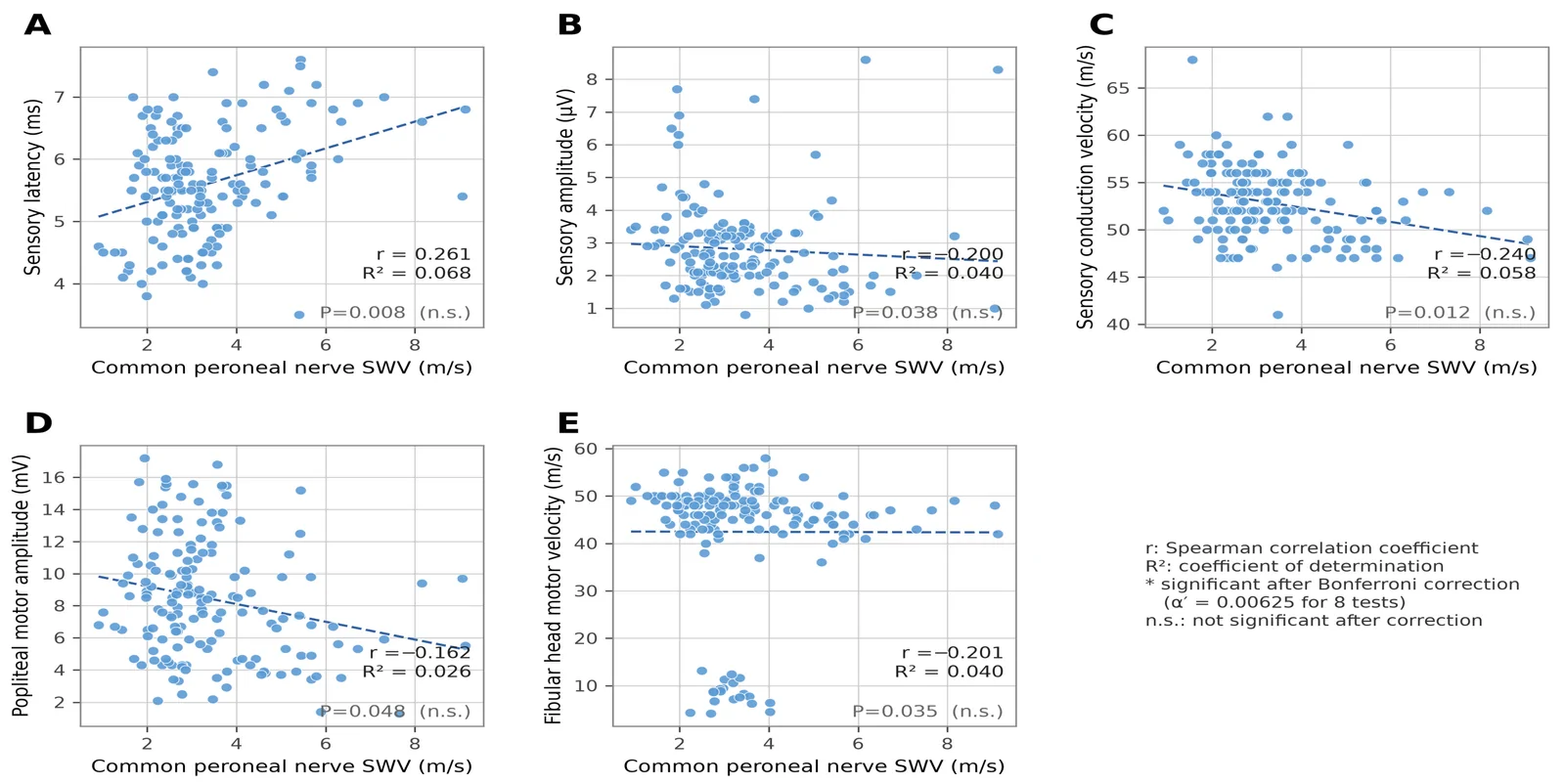
Correlation analysis between common peroneal nerve SWV and nerve electrophysiological parameters. (**A**) Positive correlation between SWV and sensory nerve conduction (SCV) latency (ms) of the common peroneal nerve. (**B**) Negative correlation between SWV and SCV peak-to-peak amplitude (μV) of the common peroneal nerve. (**C**) Negative correlation between SWV and SCV conduction velocity (m/s) of the common peroneal nerve. (**D**) Negative correlation between SWV and motor nerve conduction (MCV) popliteal fossa peak-to-peak amplitude (mV) of the common peroneal nerve. (**E**) Negative correlation between SWV and MCV fibular head conduction velocity (m/s) of the common peroneal nerve. Light blue dots represent individual observations, and the dark blue dashed line denotes the linear regression fit; asterisks (*) indicate significance after Bonferroni correction (α′ = 0.00625 for 8 tests), whereas n.s. denotes non-significant correlations, as defined in the figure legend.

For the common peroneal nerve, Spearman rank correlation analysis (with mixed-effects adjusted *p* values) showed that SWV was negatively correlated with its sensory nerve conduction peak-to-peak amplitude (*r* = −0.200, *p* = 0.038), sensory conduction velocity (*r* = −0.240, *p* = 0.012), motor nerve conduction popliteal fossa peak-to-peak amplitude (*r* = −0.162, *p* = 0.048), and fibular head conduction velocity (*r* = −0.201, *p* = 0.035). It was also positively correlated with sensory nerve conduction latency (*r* = 0.261, *p* = 0.008) (Figure 4). However, after Bonferroni correction (α’ = 0.00625), none of these correlations remained statistically significant (all |*r*| < 0.27, R^2^ < 0.07), indicating that the associations between common peroneal nerve SWV and electrophysiological parameters were statistically detectable but very weak in magnitude, with each SWV parameter explaining less than 7% of the variance in NCS parameters.

### 3.5. Diagnostic Efficacy of Ultrasound Elastography for PD Complicated with PN

**Figure 5:**
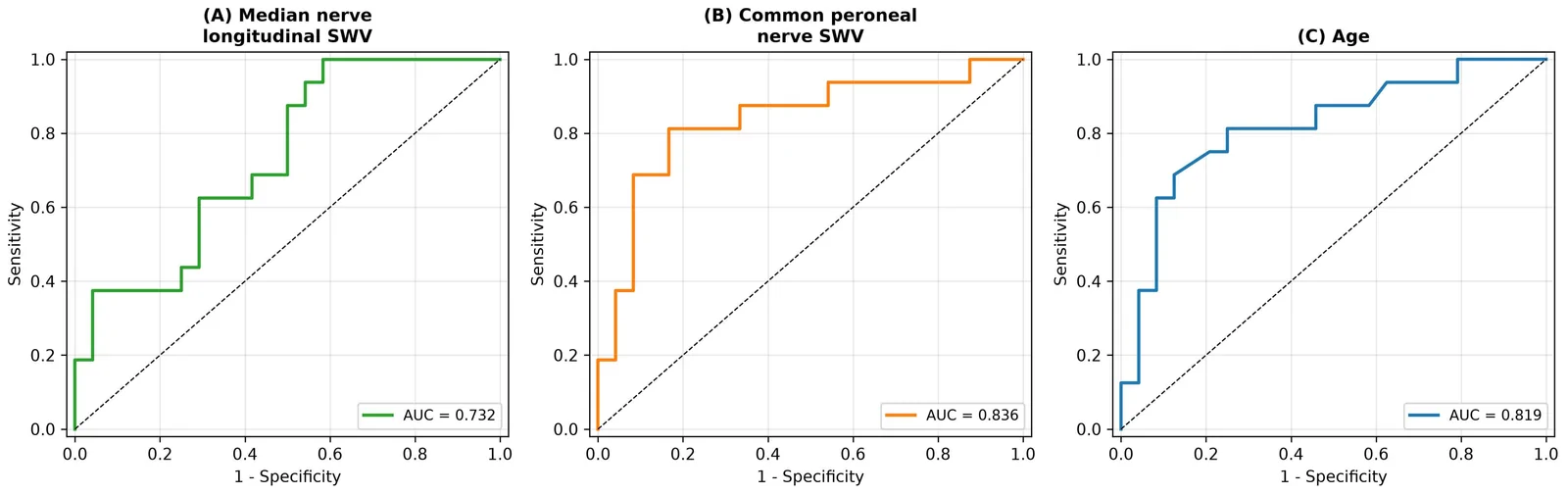
Diagnostic efficacy of univariate analysis for detecting PN in PD patients. (**A**) ROC curve of median nerve longitudinal SWV for diagnosing PD-PN; (**B**) ROC curve of common peroneal nerve SWV for diagnosing PD-PN; (**C**) ROC curve of age for diagnosing PD-PN.

Univariate ROC curve analysis was performed at the subject level in 40 PD patients (16 PD-PN vs. 24 PD-alone). The AUC of median nerve longitudinal SWV for diagnosing PD-PN was 0.732 (95% CI 0.558–0.875), with a sensitivity of 100.00% and a specificity of 41.67%. The AUC of common peroneal nerve SWV was 0.836 (95% CI 0.688–0.962), with a sensitivity of 81.25% and a specificity of 83.33%. The AUC of age was 0.819 (95% CI 0.670–0.949), with a sensitivity of 68.75% and a specificity of 87.50% (Figure 5). The optimal cutoff values based on the Youden index were ≥5.28 m/s for median nerve longitudinal SWV, ≥4.19 m/s for common peroneal nerve SWV, and ≥65.5 years for age.

Variables that showed statistically significant differences in univariate analysis (age, median nerve longitudinal SWV, common peroneal nerve SWV) were entered into a multivariate binary logistic regression model at the subject level (40 PD patients: 16 PD-PN vs. 24 PD-alone). Age and common peroneal nerve SWV emerged as independent risk factors, while median nerve longitudinal SWV showed a positive but non-significant association (*p* = 0.128) in the multivariable model (Table 2). As a sensitivity analysis, Firth-penalized logistic regression [21] yielded consistent results (median nerve longitudinal SWV: B = 0.511, OR = 1.667, *p* = 0.173), confirming that the non-significance was not an artifact of standard maximum-likelihood estimation in a small sample. Median nerve longitudinal SWV was retained in the model because (i) its inclusion improved the AUC from 0.914 to 0.938 and yielded a lower Akaike information criterion (AIC; 33.8 vs. 34.8), with a marginally significant likelihood-ratio test (*p* = 0.082); (ii) its OR (2.003) suggested a clinically meaningful positive association, albeit with a wide CI (0.819–4.900) reflecting limited statistical power; and (iii) upper-limb nerve involvement is a recognized feature of PD-associated PN. The multivariable model included 3 predictors with 16 events (PD-PN cases), yielding an EPV of approximately 5.3, below the conventional minimum of 10 [20]; the risk of overfitting is acknowledged.

**Figure 6:**
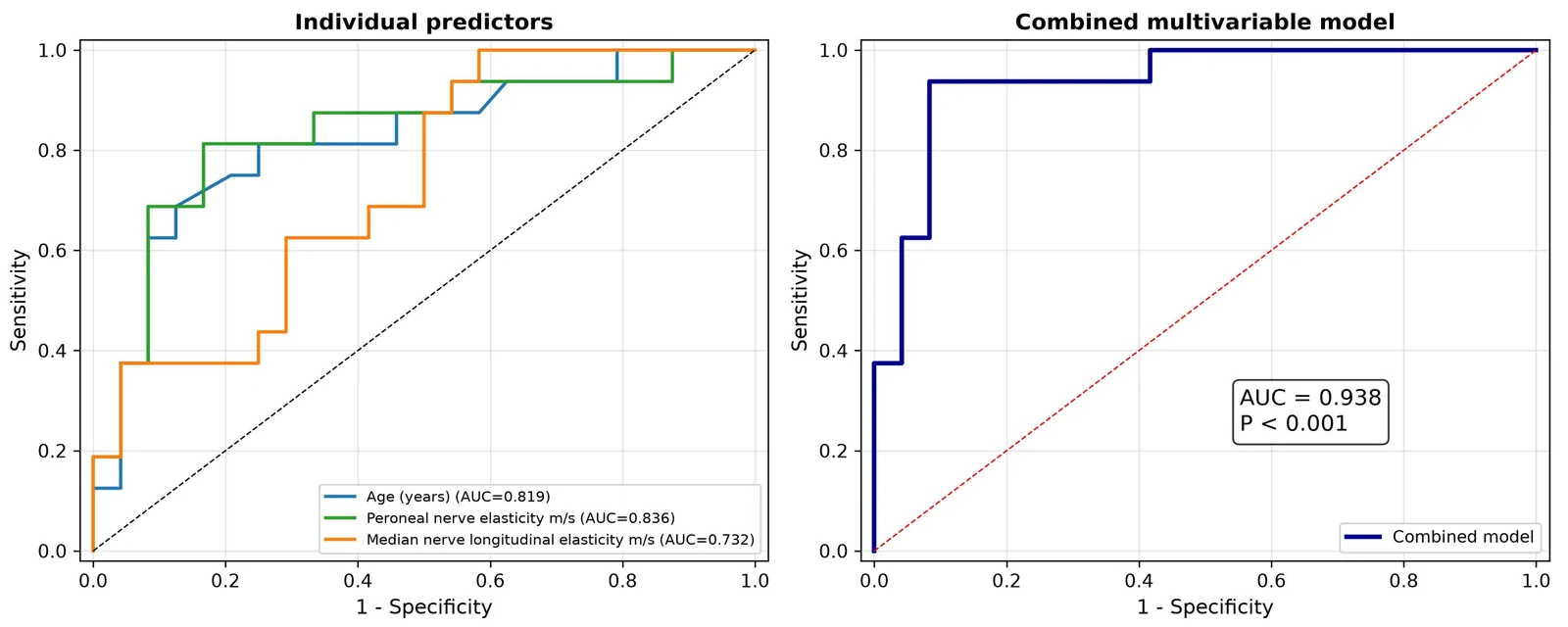
ROC curves for diagnosing PD-PN. (**Left**) panel Individual ROC curves for age, median nerve longitudinal SWV, and common peroneal nerve SWV. (**Right**) panel ROC curve of the combined multivariable logistic regression model incorporating all three predictors (AUC = 0.938, 95% CI 0.869–1.000). The dashed diagonal line in each panel is the no-discrimination (chance) reference line corresponding to an area under the curve of 0.5.

A combined diagnostic prediction model was established using the enter method with age, median nerve longitudinal SWV, and common peroneal nerve SWV. The AUC of this model for diagnosing PD-PN among PD patients reached 0.938 (95% CI 0.869–1.000), with a sensitivity of 93.75% and a specificity of 91.67% (Figure 6). Bootstrap internal validation (1000 iterations) [18] yielded an optimism-corrected AUC of 0.906 (95% CI 0.859–0.940), with a corrected sensitivity of 79.0% and specificity of 86.6%, indicating a modest degree of overfitting (optimism = 0.031). Calibration assessment [22] revealed a Brier score of 0.103 and a calibration slope of 1.005 (95% CI 0.724–1.254), with a non-significant Hosmer–Lemeshow test [23] (χ^2^ = 5.325, *p* = 0.150), indicating good calibration. DCA [19] demonstrated positive net benefit across threshold probabilities of 0.05–0.95, with a net benefit of 0.331 at the optimal threshold of 0.47. The calibration plot, DCA curve, and bootstrap internal validation figure are provided as Figures S1–S3.

The logistic regression equation for the combined model was Logit(P) = −21.852 + 0.194 × Age + 0.695 × Median nerve longitudinal SWV (m/s) + 1.203 × Common peroneal nerve SWV (m/s), where P(PD-PN) = 1/[1 + exp(−Logit(P))]. A probability cutoff of ≥0.47 (based on the Youden index) provided optimal diagnostic performance (sensitivity = 93.75%, specificity = 91.67%). The diagnostic performance and optimal cutoff values of all single parameters and the combined model are summarized in Table 3.

## 4. Discussion

The present study demonstrated that the longitudinal SWV of the median nerve and the SWV of the common peroneal nerve were significantly elevated in PD patients with PN compared with those in the PD-alone and healthy control groups. These SWV values showed weak-to-moderate correlations with nerve conduction parameters (significant only for median nerve motor conduction latencies after Bonferroni correction), suggesting that SWV may serve as a reliable, non-invasive, quantitative biomarker for evaluating PN in PD patients. Multivariate analysis identified age and common peroneal nerve SWV as independent risk factors for PN in PD patients (OR = 1.214 and 3.329, respectively), while median nerve longitudinal SWV showed a positive but non-significant association (OR = 2.003, *p* = 0.128), likely reflecting limited statistical power owing to the small sample size. The prediction model incorporating age, median nerve longitudinal SWV, and common peroneal nerve SWV achieved an AUC of 0.938 (95% CI 0.869–1.000; bootstrap-corrected AUC = 0.906), indicating that the combined application of multi-parameter SWE can effectively enhance diagnostic accuracy for PD-associated PN.

The pathological mechanisms underlying PD complicated with PN are multifactorial, involving abnormal α-synuclein deposition in peripheral nerves, activation of inflammatory cascades, vitamin B12 deficiency, and long-term levodopa therapy [10,11]. Evidence from skin biopsy studies has confirmed phosphorylated α-synuclein deposition in cutaneous nerve fibers of PD patients, which correlates with intraepidermal nerve fiber density (IENFD) reduction [15]. These pathological processes may damage both axons and myelin sheaths, leading to structural disorganization, endoneurial fibrosis, and increased nerve stiffness, thereby providing the pathophysiological rationale for SWE-based diagnosis of PN through quantitative assessment of tissue elasticity. In the present study, SWV values in the PD-PN group were significantly elevated, consistent with findings reported in diabetic peripheral neuropathy [12], suggesting that increased nerve stiffness may represent a common biomechanical manifestation of peripheral nerve injury across diverse etiologies.

The clinical diagnosis of PN in PD patients remains challenging. A recent systematic review and meta-analysis reported a pooled prevalence of PN in PD of approximately 35%, with large-fiber neuropathy predominating [3]. Notably, recent evidence indicates that SFN may precede dopaminergic treatment initiation, suggesting that peripheral nerve involvement may occur early in the disease course [8]. PN has been recognized as a marker of a more severe disease phenotype in PD [24]. These findings underscore the need for sensitive diagnostic tools capable of detecting both large- and small-fiber pathology. The high diagnostic performance of SWE observed in the present study supports its potential role in bridging this clinical gap, particularly given its non-invasive nature and excellent reproducibility.

Our results revealed that longitudinal SWV of the median nerve possessed significant diagnostic value, whereas cross-sectional SWV did not reach statistical significance (control: 4.187 ± 1.105, PD-alone: 5.476 ± 1.854, PD-PN: 6.712 ± 1.723 m/s; *p* = 0.062). This discrepancy is closely related to the unique anatomical and biomechanical characteristics of peripheral nerves. Peripheral nerves are composed of numerous nerve fiber bundles aligned predominantly along the longitudinal axis, and their anisotropic mechanical properties dictate that tissue stiffness measured in the longitudinal direction more faithfully reflects the structural integrity of nerve fascicles. In contrast, cross-sectional stiffness measurements are more susceptible to interference from perineural connective tissue, epineurial blood vessels, and adipose tissue [13,25]. Given that the pathological damage in PD-associated PN primarily involves axonal degeneration and demyelination of nerve fiber bundles, the structural alterations are biomechanically more pronounced in the longitudinal orientation, which explains why longitudinal SWV can more accurately capture pathological stiffness changes in peripheral nerves of PD patients. These findings are consistent with prior SWE studies and systematic reviews of peripheral nerve mechanics, which have consistently reported higher and more reliable shear wave velocities when the transducer is aligned parallel to the nerve fibers (longitudinal plane) rather than perpendicular to them (cross-sectional plane) [26].

The weak-to-moderate correlations between SWV and electrophysiological parameters warrant nuanced interpretation. The pathophysiological link between nerve stiffness and electrophysiological function is complex: SWE measures the biomechanical stiffness of nerve tissue (reflecting structural changes such as endoneurial fibrosis, demyelination, and axonal loss), whereas NCS assesses the electrical conductivity of large myelinated fibers. These two modalities evaluate complementary aspects of nerve pathology, and structural stiffness changes may precede or occur independently of functional electrophysiological deterioration. This partial dissociation may explain the modest correlations observed in the present study, consistent with prior SWE studies in diabetic peripheral neuropathy that have also reported weak-to-moderate correlations between SWV and NCS parameters [12,27]. It is important to distinguish statistical significance from effect size: although several correlations reached statistical significance (partly due to the number of bilateral measurements), the coefficient of determination (R^2^) indicates that SWV explains only 3.6–22.3% of the variance in individual NCS parameters, underscoring that these are weak-to-moderate associations rather than strong linear relationships.

An important limitation of the present study is that PD-PN classification relied entirely on NCS, which primarily assess large myelinated fiber (Aα and Aβ) function and cannot detect isolated SFN involving unmyelinated C fibers and thinly myelinated Aδ fibers. SFN is increasingly recognized in PD, with studies reporting reduced IENFD on skin biopsy and corneal nerve fiber loss on corneal confocal microscopy (CCM) even in patients with normal NCS [8]. By using NCS as the reference standard, we may have misclassified some patients with isolated SFN as “PD without PN,” potentially attenuating the observed group differences. Future studies should adopt a multi-modal diagnostic approach combining NCS with skin biopsy (IENFD quantification) [28], quantitative sensory testing (QST) using standardized protocols [29], and CCM [30] to achieve comprehensive assessment of both large- and small-fiber involvement in PD.

The generalizability of our findings should be considered in light of the single-center, single-platform design. Cross-platform generalizability is a known challenge in SWE research, as different ultrasound vendors use different shear wave generation and detection algorithms that can produce systematically different SWV values for the same tissue. Therefore, the specific cutoff values reported herein may not be directly transferable to other platforms, and platform-specific calibration would be necessary. While intra- and inter-observer reproducibility was good locally (ICC > 0.855), these results may not generalize to observers with different experience levels. Regarding the position of SWE in the diagnostic workflow, we envision SWE as a non-invasive, rapid first-line screening tool for PD patients at risk of PN, with positive cases referred for confirmatory NCS. SWE should not replace NCS as the diagnostic gold standard at this stage, and further multicenter, cross-platform validation is required before clinical implementation.

The non-significant findings for nerve CSA warrant nuanced interpretation. On one hand, the absence of significant CSA differences is consistent with the hypothesis that SWV detects microstructural stiffness changes (endoneurial fibrosis, demyelination, axonal loss) before overt morphological enlargement becomes measurable, as previously reported in diabetic peripheral neuropathy [12,27]. On the other hand, measurement limitations may also contribute: CSA manual tracing is subject to inter-observer variability, particularly for the flat common peroneal nerve at the fibular head, and PD-associated PN is a length-dependent diffuse process rather than a focal entrapment, which may not produce measurable CSA enlargement at the examined sites. The relative contribution of true early biological change versus measurement insensitivity cannot be definitively resolved in the present study.

Several limitations of the present study should be acknowledged. First, previous studies [14] have reported that approximately 53% of PD patients develop PN of varying severity, and SFN may be present even before the initiation of dopaminergic therapy. The present study employed NCS as the reference standard, which primarily assesses large-fiber function and has inherent limitations in detecting early-stage small-fiber pathology. Established diagnostic criteria for small-fiber neuropathy have emphasized the complementary role of skin biopsy with quantification of IENFD, CCM, and QST for comprehensive evaluation of SFN in PD [31]. Future studies should incorporate these small-fiber assessment modalities to achieve a more comprehensive evaluation of PD-associated PN and to validate SWE findings against histological evidence. Second, this study adopted a single-center, single-platform cross-sectional design with a relatively limited sample size, which precluded dynamic observation of the longitudinal relationship between SWV parameters and PD duration and severity, and did not permit adequately powered stratified analyses of potential confounding factors such as cumulative levodopa exposure, nutritional status, and metabolic comorbidities. Although our exclusion criteria explicitly eliminated patients with diabetes mellitus, thyroid dysfunction, renal insufficiency, and other metabolic causes of neuropathy, thereby reducing metabolic confounding, we acknowledge that disease duration, levodopa equivalent daily dose (LEDD), serum vitamin B12, homocysteine, and methylmalonic acid levels were not systematically collected, and subclinical B12 deficiency cannot be entirely ruled out [5,14]. Age, which is strongly correlated with disease duration and levodopa exposure in PD cohorts, was included as a covariate and partially accounts for these effects; however, residual confounding by these unmeasured variables remains possible. Additionally, key PD-associated clinical measures including Unified Parkinson’s Disease Rating Scale (UPDRS) scores, Hoehn and Yahr stage, and cognitive status (Montreal Cognitive Assessment [MoCA]/Mini-Mental State Examination [MMSE]) were not systematically recorded, which limits our ability to determine whether nerve stiffness changes specifically reflect neuropathy or overall disease burden. We note, however, that the diagnostic model compares PD-PN versus PD-alone patients (both groups have PD), so the observed SWE differences cannot be attributed to PD burden per se. Future prospective studies should systematically collect these clinical variables and test their association with SWE parameters. Large-scale, multicenter, prospective longitudinal cohort studies are warranted to validate these findings and to establish the temporal trajectory of SWV changes in relation to PD progression. Third, the absence of histological correlation limits definitive conclusions regarding the specific pathological substrates underlying the observed stiffness changes. Fourth, the PD-PN group was significantly older than the other groups, and although ANCOVA adjustment confirmed that SWV remained significantly associated with PN after controlling for age, the observational design cannot fully exclude residual confounding by age or age-related factors. Fifth, the multivariable logistic regression model had a low EPV ratio (≈5.3), which increases the risk of overfitting; bootstrap internal validation [18] yielded a corrected AUC of 0.906 (vs. apparent AUC of 0.938), and external validation in larger independent cohorts is needed before clinical application. Furthermore, median nerve longitudinal SWV did not reach statistical significance in the multivariable model (*p* = 0.128), which likely reflects the limited sample size and low EPV rather than a true absence of effect; its OR of 2.003 and the AUC improvement upon inclusion support its retention. The use of standard logistic regression with forced entry, while avoiding the instability of stepwise variable selection in small datasets, may still benefit from penalized regression approaches (ridge or LASSO) in larger cohorts to further reduce overfitting and improve coefficient stability. Sixth, the original analysis pooled bilateral measurements, which can inflate Type I error due to within-subject correlation; the revised analyses use per-subject averages and mixed-effects models to address this issue. The original bilateral pooled analysis is provided as a sensitivity analysis in the Supplementary Materials, with an explicit note that this approach does not account for with-in-subject correlation and should be interpreted with caution.

## 5. Conclusions

HFUS combined with SWE for the measurement of longitudinal SWV of the median nerve and SWV of the common peroneal nerve demonstrates favorable diagnostic performance for PD complicated with PN, with strong inter-observer and intra-observer reproducibility. The prediction model integrating age with multi-nerve SWV parameters can further improve diagnostic efficiency, providing a reliable, non-invasive imaging tool to support the early identification of PN in PD patients. Future multicenter longitudinal studies incorporating comprehensive small-fiber evaluation and histological validation are warranted to confirm the clinical utility of SWE in this population and to elucidate the temporal dynamics of nerve stiffness changes across the disease course.

## Figures and Tables

**Table 1:** Consistency test results of ultrasound parameter measurements.

Parameters	Intra-Observer ICC (95% CI)	Inter-Observer ICC (95% CI)
Median nerve CSA	0.865 (0.710–0.935)	0.855 (0.694–0.931)
Median nerve cross-sectional SWV	0.882 (0.737–0.945)	0.870 (0.707–0.940)
Median nerve longitudinal SWV	0.892 (0.774–0.948)	0.864 (0.715–0.935)
Common peroneal nerve CSA	0.892 (0.773–0.948)	0.872 (0.729–0.939)
Common peroneal nerve SWV	0.895 (0.782–0.950)	0.883 (0.754–0.944)

Note: CSA, cross-sectional area; SWV, shear wave velocity; ICC, intraclass correlation coefficient; CI, confidence interval.

**Table 2:** Multivariate logistic regression analysis of independent risk factors for PD complicated with PN.

Parameters	B	SE	Wald Value	*p* Value	OR	95% CI Lower	95% CI Higher
Age	0.194	0.078	6.142	0.013	1.214	1.041	1.414
Median nerve longitudinal SWV	0.695	0.456	2.318	0.128	2.003	0.819	4.900
Common peroneal nerve SWV	1.203	0.481	6.241	0.013	3.329	1.296	8.553

Note: SE, standard error; OR, odds ratio; CI, confidence interval; SWV, shear wave velocity; PD, Parkinson’s disease; PN, peripheral neuropathy. All regression coefficients were verified to three decimal places for internal consistency (exp(B) = OR, and 95% CI = exp(B ± 1.96 × SE)).

**Table 3:** Diagnostic performance and optimal cutoff values (Youden index) of single parameters and the combined model for diagnosing PD-PN.

Parameter	AUC (95% CI)	Optimal Cutoff	Youden Index	Sensitivity	Specificity
Median nerve longitudinal SWV	0.732 (0.558–0.875)	≥5.28 m/s	0.417	100.00%	41.67%
Common peroneal nerve SWV	0.836 (0.688–0.962)	≥4.19 m/s	0.646	81.25%	83.33%
Age	0.819 (0.670–0.949)	≥65.5 years	0.562	68.75%	87.50%
Combined model	0.938 (0.869–1.000)	Probability ≥ 0.47	0.854	93.75%	91.67%

## Data Availability

The raw experimental data cannot be publicly shared due to ethical and privacy restrictions. The raw data supporting the conclusions of this article will be made available by the authors on request.

## Supplementary Materials

The following supporting information can be downloaded at https://www.mdpi.com/article/10.3390/brainsci16090988/s1. Supplementary Results, including (S1) a non-parametric Kruskal–Wallis sensitivity analysis, (S2) a bilateral-pooled group-comparison sensitivity analysis, and (S3) a bilateral-pooled diagnostic model sensitivity analysis (Table S1–S3), and Figure S1–S3 (a calibration plot, a decision curve analysis, and a bootstrap internal validation figure, all based on the subject-level PD-only dataset).

## Abbreviations

The following abbreviations are used in this manuscript:

**Abbreviation**	**Full Name**
AAN	American Academy of Neurology
AIC	Akaike information criterion
ANCOVA	Analysis of covariance
AUC	Area under the curve
CCM	Corneal confocal microscopy
CI	Confidence interval
CMAP	Compound muscle action potential
CSA	Cross-sectional area
DCA	Decision curve analysis
EPV	Events per variable
HFUS	High-frequency ultrasound
ICC	Intraclass correlation coefficient
IENFD	Intraepidermal nerve fiber density
LEDD	Levodopa equivalent daily dose
MCV	Motor nerve conduction velocity
MMSE	Mini-Mental State Examination
MoCA	Montreal Cognitive Assessment
NCS	Nerve conduction study
OR	Odds ratio
PD	Parkinson’s disease
PD-alone	Parkinson’s disease without peripheral neuropathy
PD-PN	Parkinson’s disease complicated with peripheral neuropathy
PN	Peripheral neuropathy
QST	Quantitative sensory testing
ROC	Receiver operating characteristic
ROI	Region of interest
SCV	Sensory nerve conduction velocity
SFN	Small-fiber neuropathy
SNAP	Sensory nerve action potential
STROBE	Strengthening the Reporting of Observational Studies in Epidemiology
SWE	Shear wave elastography
SWV	Shear wave velocity
UPDRS	Unified Parkinson’s Disease Rating Scale
