# Supplementary material for: Application of Shear Wave Elastography in Assessing Peripheral Neuropathy in Parkinson’s Disease
Source: Brain Sci. 2026 Sep 17;16(9):988. doi: 10.3390/brainsci16090988 (PMC13604399; doi:10.3390/brainsci16090988)
Supplement: Supplementary file 1 [file brainsci-16-00988-s001.zip › brainsci-4503989-supplementary.pdf]

brain sciences

MDPI

Application of Shear Wave Elastography in Assessing Peripheral Neuropathy in Parkinson's Disease

Supplementary Materials

Supplementary Results: Sensitivity Analyses

To verify the robustness of the primary per-subject analyses, three sensitivity analyses were performed: (1) a non-parametric Kruskal–Wallis analysis that does not assume normality; (2) the original analysis that pools bilateral (left and right) measurements for group comparisons; and (3) a bilateral-pooled diagnostic model (ROC and logistic regression) for comparison with the primary subject-level diagnostic model. All diagnostic model analyses (ROC, logistic regression, bootstrap internal validation, calibration, and decision curve analysis) in the primary analysis were performed at the subject level using PD patients only (16 PD-PN vs. 24 PD-alone), excluding healthy controls. Figures S1–S3 present the calibration plot, decision curve analysis, and bootstrap internal validation of this subject-level PD-only diagnostic model.

Supplementary Result 1. Non-parametric Kruskal–Wallis sensitivity analysis

Because the Shapiro–Wilk test has limited statistical power to detect deviations from normality in the smallest subgroup (PD-PN, n = 16), the three-group comparisons of nerve SWV were repeated using the non-parametric Kruskal–Wallis test on per-subject averages. The results were fully consistent with the parametric (ANOVA/ANCOVA) findings: both median nerve longitudinal SWV (H = 37.86, P < 0.001) and common peroneal nerve SWV (H = 23.04, P < 0.001) differed significantly among the control, PD-alone, and PD-PN groups (Table S1), confirming that the parametric inference does not depend on the normality assumption.

Table S1. Non-parametric Kruskal–Wallis sensitivity analysis of nerve SWV among the three groups (per-subject averages, df = 2).

Parameter	Kruskal–Wallis H	P value
Median nerve longitudinal SWV	37.86	< 0.001
Common peroneal nerve SWV	23.04	< 0.001

Supplementary Result 2. Bilateral-pooled sensitivity analysis

The primary analysis used the per-subject average of the bilateral measurements as the analysis unit to avoid inflation of Type I error by within-subject (left–right) correlation. As a sensitivity analysis, the original approach pooling both sides (160 nerve-level observations from 80 subjects) was retained. This approach does not account for within-subject correlation and should therefore be interpreted with caution; nevertheless, the conclusions were unchanged, with both parameters remaining highly significant (Table S2).

Table S2. Bilateral-pooled one-way ANOVA of nerve SWV among the three groups (160 bilateral observations; this analysis does not adjust for within-subject correlation and is shown for sensitivity purposes only).

Parameter	One-way ANOVA F	P value
Median nerve longitudinal SWV	37.23	< 0.001

Parameter	One-way ANOVA F	P value
Common peroneal nerve SWV	28.92	< 0.001

Supplementary Result 3. Bilateral-pooled diagnostic model sensitivity analysis

As a sensitivity analysis, the combined diagnostic model was also fitted using bilateral-pooled data (80 nerve-level observations from 40 PD patients: 32 PD-PN sides vs. 48 PD-alone sides). This approach does not account for within-subject (left–right) correlation and should be interpreted with caution. The bilateral-pooled model yielded an AUC of 0.875 (sensitivity 75.00%, specificity 91.67%), which is lower than the subject-level AUC of 0.938 (sensitivity 93.75%, specificity 91.67%), confirming that the subject-level analysis provides more reliable and conservative diagnostic performance estimates. The univariate results are summarized in Table S3.

Table S3. Diagnostic performance of the combined model for diagnosing PD-PN: comparison between subject-level analysis (primary) and bilateral-pooled analysis (sensitivity). The bilateral-pooled analysis uses 80 nerve-level observations from 40 PD patients (32 PD-PN sides vs. 48 PD-alone sides) and does not account for within-subject correlation; it is shown for sensitivity comparison only.

Parameter	Subject-level AUC (95% CI)	Subject-level Sensitivity	Subject-level Specificity	Bilateral-pooled AUC	Bilateral-pooled Sensitivity	Bilateral-pooled Specificity
Age	0.819 (0.670–0.949)	68.75%	87.50%	0.811	75.00%	83.33%
Median nerve longitudinal SWV	0.732 (0.558–0.875)	100.00%	41.67%	0.685	87.50%	50.00%
Common peroneal nerve SWV	0.836 (0.688–0.962)	81.25%	83.33%	0.782	84.38%	66.67%
Combined model	0.938 (0.869–1.000)	93.75%	91.67%	0.875	75.00%	91.67%

Supplementary Figures

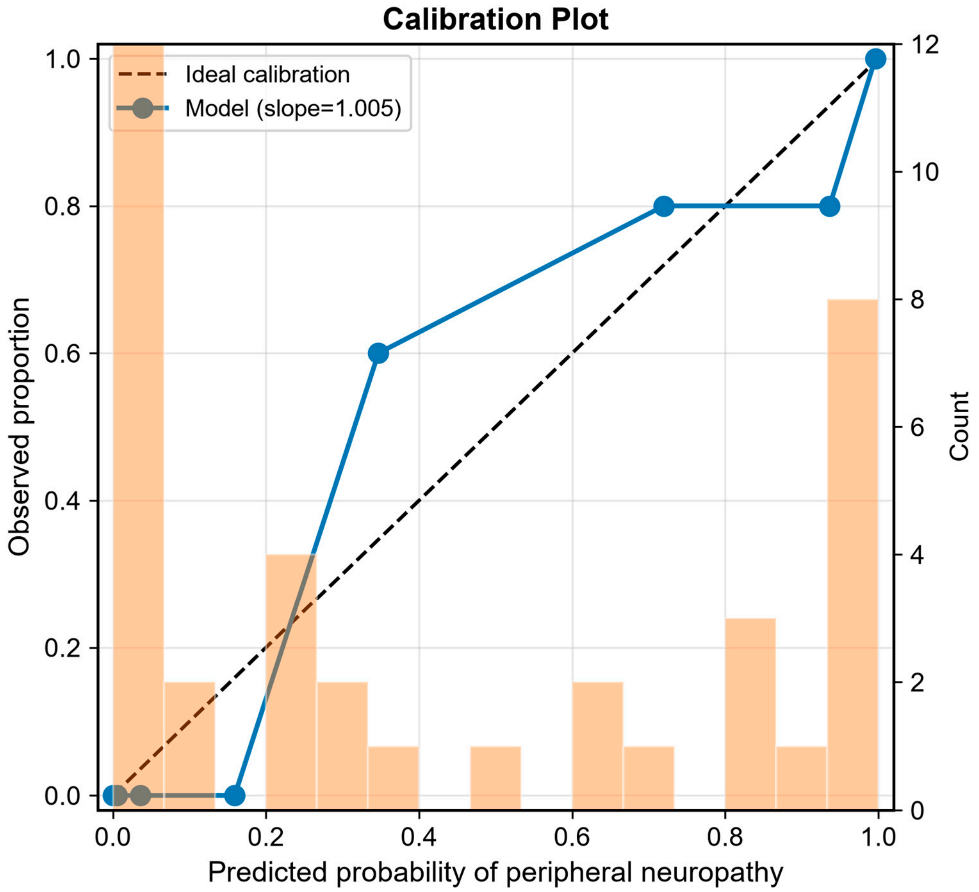

Figure S1. Calibration plot of the combined prediction model, based on 40 PD patients (16 PD-PN vs. 24 PD-alone) at the subject level (per-subject averages of bilateral measurements), excluding healthy controls. The calibration curve (blue) shows the agreement between the predicted probability of PN (x-axis) and the observed proportion (y-axis); the dashed diagonal denotes ideal calibration and the orange points denote octile-grouped observed frequencies. The calibration slope was 1.005 (95% CI 0.724–1.254) and the Brier score was 0.103, with a non-significant Hosmer–Lemeshow test ($\chi^2 = 5.325$, $P = 0.150$), indicating good calibration.

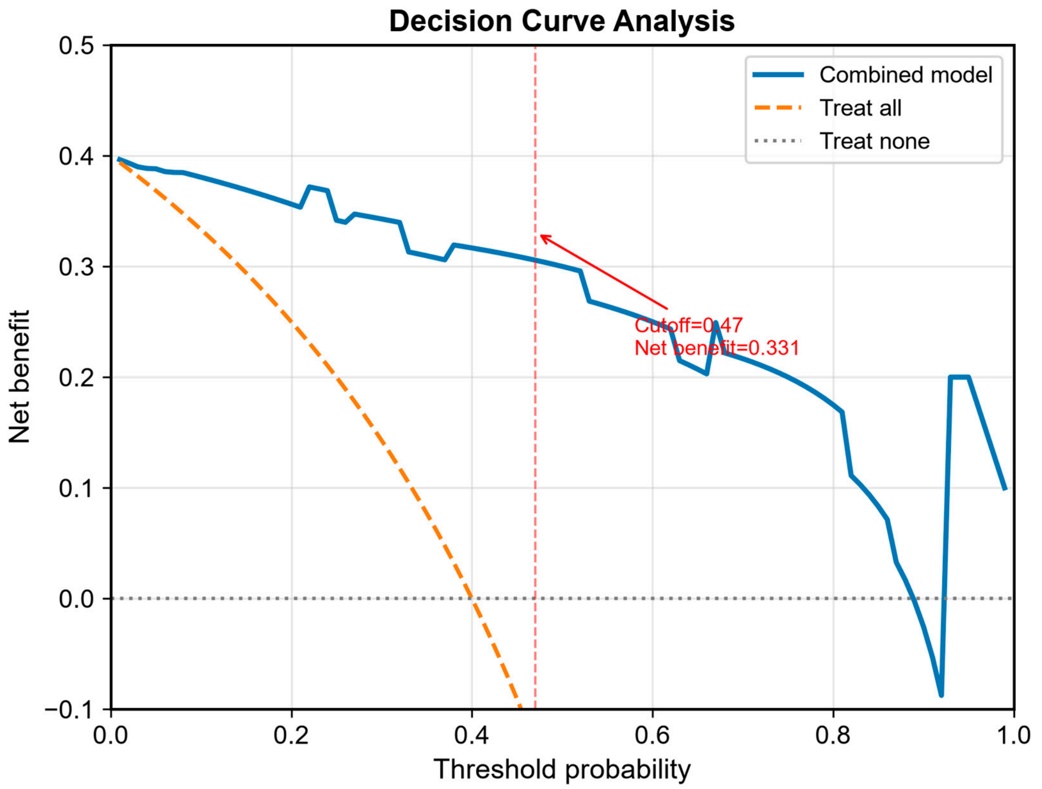

Figure S2. Decision curve analysis (DCA) of the combined prediction model, based on 40 PD patients (16 PD-PN vs. 24 PD-alone) at the subject level, excluding healthy controls. Net benefit (y-axis) is plotted against threshold probability (x-axis). The blue line is the combined model, the orange dashed line the "treat-all" strategy, and the dotted line the "treat-none" strategy. The combined model provided a higher net benefit than both reference strategies across threshold probabilities of approximately 0.05–0.95; at the optimal probability cutoff of 0.47 the net benefit was 0.331.

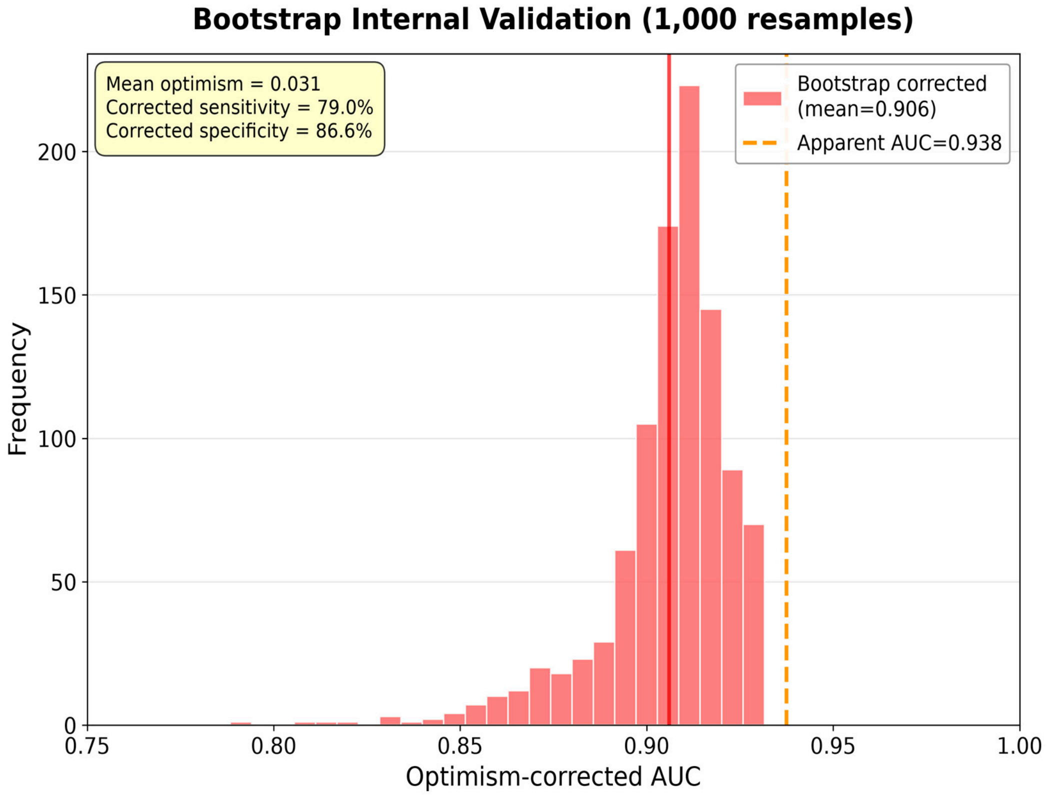

Figure S3. Bootstrap internal validation of the combined prediction model, based on 40 PD patients (16 PD-PN vs. 24 PD-alone) at the subject level, excluding healthy controls. Distribution of the optimism-corrected area under the ROC curve (AUC) across 1,000 bootstrap resamples (red solid line, mean = 0.906), with the apparent AUC of 0.938 shown for comparison (orange dashed line); the mean optimism was 0.031, with a corrected sensitivity of 79.0% and specificity of 86.6%, indicating a modest degree of overfitting.
